# Three-dimensional surface lattice plasmon resonance effect from plasmonic inclined nanostructures via one-step stencil lithography

**DOI:** 10.1515/nanoph-2023-0755

**Published:** 2024-01-12

**Authors:** Tae-In Jeong, Sehyeon Kim, San Kim, Minchan Shin, Alexander Gliserin, Tae Young Kang, Kyujung Kim, Seungchul Kim

**Affiliations:** Department of Cogno-Mechatronics Engineering, College of Nanoscience and Nanotechnology, Pusan National University, Busan 46241, Republic of Korea; Department of Optics and Mechatronics Engineering, College of Nanoscience and Nanotechnology, Pusan National University, Busan 46241, Republic of Korea

**Keywords:** surface lattice plasmon resonance, stencil lithography, TEM grid, nanophotonics

## Abstract

Plasmonic nanostructures allow the manipulation and confinement of optical fields on the sub-wavelength scale. The local field enhancement and environmentally sensitive resonance characteristics provided by these nanostructures are of high importance for biological and chemical sensing. Recently, surface lattice plasmon resonance (SLR) research has attracted much interest because of its superior quality factor (*Q*-factor) compared to that of localized surface plasmon resonances (LSPR), which is facilitated by resonant plasmonic mode coupling between individual nanostructures over a large area. This advantage can be further enhanced by utilizing asymmetric 3D structures rather than low-height (typically height < ∼60 nm) structure arrays, which results in stronger coupling due to an increased mode volume. However, fabricating 3D, high-aspect ratio, symmetry-breaking structures is a complex and challenging process even with state-of-the-art fabrication technology. Here, we report a plasmonic metasurface of 3D inclined structures produced via commercial TEM grid–based stencil lithography with a *Q*-factor of 101.6, a refractive index sensitivity of 291 nm/RIU, and a figure of merit (FOM) of 44.7 in the visible wavelength range at a refractive index of 1.5 by utilizing the 3D SLR enhancement effect, which exceeds the performance of most LSPR systems (*Q* < ∼10). The symmetry-breaking 3D inclined structures that are fabricated by electron beam evaporation at an angle increase the polarizability of the metasurface and the directionality of the diffractively scattered radiative field responsible for SLR mode coupling. Additionally, we explore the role of spatial coherence in facilitating the SLR effect and thus a high-*Q* plasmonic response from the nanostructures. Our work demonstrates the feasibility of producing 3D inclined structure arrays with pronounced SLR enhancement for high biological sensitivity by utilizing the previously unexplored inclined stencil lithography, which opens the way to fabricate highly sensitive plasmonic metasurfaces with this novel simple technique.

## 1 Introduction

Light interaction with metallic nanostructures can induce the collective oscillation of electrons called surface plasmons (SPs) at the resonance condition [1], [2]. The tailored design of nanostructures allows utilizing the characteristics of SPs including the manipulation and confinement of optical fields on the sub-wavelength scale, which has great potential for biological and chemical detection due to the large local field enhancement and environmental sensitivity of the plasmonic resonance conditions [3], [4], [5]. However, the relatively broad spectral width and insufficient field enhancement of the localized surface plasmon resonance (LSPR) around the nanostructure may significantly limit many potential applications [6], [7], [8]. The electromagnetic coupling within periodically arranged nanostructure arrays can be used to overcome this limitation of a low quality-factor (*Q*-factor) of the LSPR [9], [10]. The polarizability of a plasmonic nanostructure enables it to act like a dipole antenna and scatter an incoming radiative field via far-field coupling of the LSPR [11], [12]. The scattered plasmonic fields from each structure are in phase with each neighbor within the array when the wavelength of the incident light is comparable with the spatial period of the structure array, which enhances the resonance in the neighboring structures [13], [14], [15]. The diffractively coupled LSPR is called surface lattice plasmon resonance (SLR) and can lead to strong field enhancement and an increased *Q*-factor [16], [17], [18]. The utilization of SLR structure arrays for biosensing applications has great advantages because of the large coupling field area compared to LSPR, and the environmentally sensitive plasmonic resonance condition matches well with the refractive index change associated with biological binding events when the target biological molecules react with receptors [19]–[22]. The recent SLR research has been focused on increasing the sensitivity of the system, and ultrahigh *Q*-factor research has been reported using the electron-beam (E-beam) lithography method to fabricate low-height (typically height < ∼60 nm) structure arrays to induce scattered radiative coupling [23]–[26]. Increasing the SLR mode coupling volume can be an efficient method to enhance the sensitivity and can be achieved by utilizing the multi-mode plasmonic resonance of 3D plasmonic structures [27]–[30]. However, despite the state-of-the-art fabrication technique of E-beam lithography, the fabrication of 3D, high-aspect ratio, and symmetry breaking nanostructures is very complex and challenging, because the electron beam is typically aligned perpendicular to the surface of the substrate, which does not allow patterning electron beam resist into vertically asymmetric structures [31]–[34]. In this work, a novel method for fabricating inclined plasmonic nanostructure arrays via one-step nanostencil lithography is demonstrated, which is used to create metasurfaces with an enhanced 3D SLR effect. The 3D geometry of the inclined nanostructures induces a large mode volume of the SLR, which increases the sensitivity of our system. The fabrication method is a combination of commercial TEM grid–based stencil lithography with an angle bracket, where the geometry of the structure is controlled by the evaporation time and angle, which allows tailoring the optical properties by changing the polarizability of the structures. In particular, we demonstrate switching characteristics of the measured reflection spectra via linear polarization change. The diffractive coupling of the LSPR within the nanostructure array is supported by Maxwell equation based numerical calculations and experimental analysis of the effect of spatial coherence on the coupling. We demonstrate a *Q*-factor of 101.6, a refractive index sensitivity of 291 nm/RIU, and a figure of merit (FOM) of 44.7 in the visible wavelength range through analysis of the resonance dip wavelength when changing the refractive index environment between 1.4 and 1.5, which is approximately a 10 times higher sensitivity compared to most LSPR systems and similar to or exceeding other SLR systems (Supplementary Table 1). These results signify a clear potential of our SLR system and fabrication technique for biomolecular detection applications.

## 2 Results and discussion

### 2.1 Principle of the lattice plasmon resonance effect from a 3D inclined structure array

**Figure 1:**
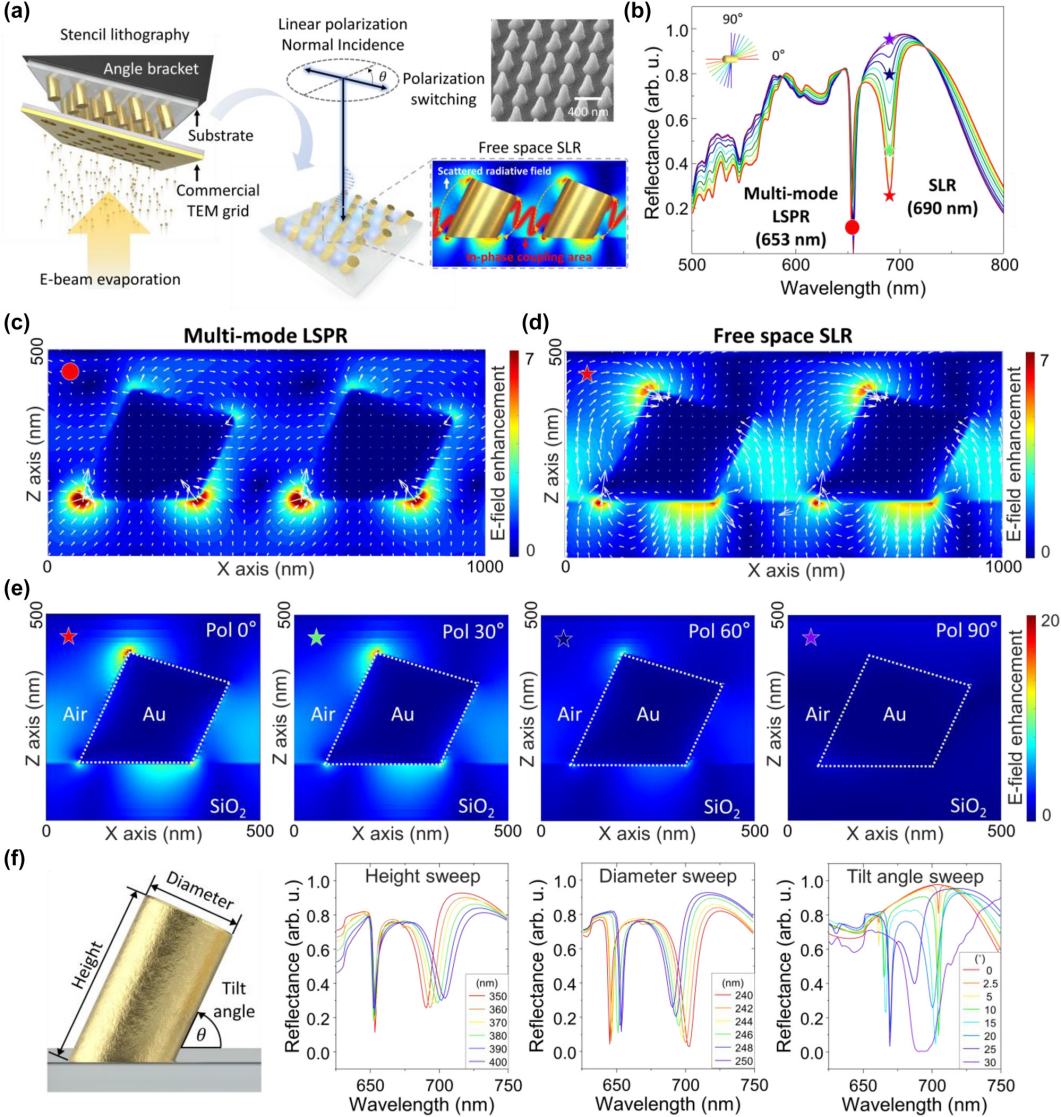
The principle of the SLR effect at a 3D inclined structure array. (a) Schematic of the lattice plasmonic effect at a 3D inclined structure array, which is fabricated with commercial TEM grid–based one-step stencil lithography. Here, diameter = 250 nm, height = 350 nm, and periodicity = 500 nm are used. The blue shaded region illustrates the electric field, indicating the SLR mode between neighboring structures; the inset shows a SEM image of the fabricated structure array (upper) and the interaction between neighboring inclined plasmonic structures (lower). (b) Reflection spectra as a function of linear polarization change, the top-left schematic shows the relation of the polarization direction with the direction of inclination of the structure. (c) Electric field profile and electric vector field flow for the polarization in-sensitive wavelength region indicated in (b) (red circle) and (d) for the polarization-sensitive wavelength region (red star). (e) Electric field profile for each incident linear polarization state (0°: red star, 30°: green star, 60°: dark blue star, 90°: purple star), as indicated in (b). (f) Reflection spectra for geometry changes of the structure (height, diameter, tilt angle).

The 3D inclined plasmonic structure array was fabricated by commercial TEM grid–based one-step stencil lithography (Figure 1a). The symmetry-breaking geometry of the inclined structures is realized by employing the E-beam evaporation method at an angle via an angle bracket attached to the sample substrate, and which enable to have polarization switchable characteristic in each polarization of light [35]. SLR occurs when the linear polarization of light at normal incidence is parallel with the inclination direction of the structure. A plasmonic multi-mode is generated at the 3D inclined structure, which induces a large mode volume of the SLR (Figure 1a inset, red solid lines) between neighboring structures when LSPR of around structure and the scattered radiative fields are in-phase. A pillar diameter of 250 nm and a periodicity of 500 nm in a hexagonal array were selected to place the SLR wavelength in the visible range.

The numerical calculation was conducted with the finite-difference time-domain (FDTD, Ansys) method to understand the underlying physics of the plasmonic symmetry-breaking structure. A single unit cell in the hexagonal array was simulated using a Bloch boundary condition in the in-plane dimension to avoid phase errors at the boundary and a perfectly matched layer in the out-of-plane dimension with the broadband fixed angle source technique (BFAST) [36]. The reflectance spectra for each linear polarization state are shown in Figure 1b, which consists of polarization-sensitive (star symbol) and in-sensitive (circle symbol) resonance wavelengths. For better understanding, we explored the electric field distribution including the vector orientation around the inclined structure array for each wavelength and linear polarization state. Note that the electric vector field flow is represented by white arrows in Figure 1c and d. At the polarization in-sensitive wavelength (red circle), the electric field vector is directed toward the bottom of the tilted structure where the electric fields become localized (Figure 1c). The polarization in-sensitive characteristic at this wavelength is due to a multi-mode LSPR induced at the circular bottom of the structure, which exhibits a quadrupole surface plasmon resonance with a narrow resonance wavelength range (Figure S1a and b). On the other hand, at the polarization-sensitive wavelength with an illumination polarization parallel to the inclination direction of the structures (red star), an asymmetric multi-mode LSPR is generated at the bottom circumference of the structure (Figure S2a) with an upward pointing electric field vector (Figure 1d). The radiative coupling between the diffractively scattered illumination field and multi-mode LSPR around structures induces a large mode volume of the SLR effect with upward electric field within the periodic array between the structures (Figure 1d). As the linear polarization is changed from parallel to perpendicular with respect to the direction of the inclination of the structure, the induced multi-mode LSPR becomes weaker (Figure 1e), affecting the reflectivity at that wavelength (Figure 1b). This result shows that the symmetry-breaking geometry of the inclined structures causes the linear polarization state of the incident light to control the strength of the SLR coupling effect.

The influence of the inclined structure geometry on the optical properties of the LSPR and SLR effect is explored in Figure 1f. The reflection spectra were calculated while changing the height, diameter, and tilt angle of the structure. The resonance wavelength of the LSPR generated at the circular bottom of the structure only depends on the change of the circular bottom geometry. As a result, the LSPR resonant wavelength is unaffected by changes in the height of the structure, but a spectral redshift is observed for increases in diameter and tilt angle of the structure, which affects the geometry of the bottom circle. In contrast, the multi-mode LSPR-based SLR effect is influenced by various factors. (1) Height: As the height increases, the top LSPR region where the upward electric field vector is localized moves away from the bottom LSPR region, which causes a redshift of the SLR resonance wavelength as well as weaker coupling evident by the spectral broadening of the resonance. (2) Diameter: A diameter increase of the structure reduces the distance between neighboring structures, causing an increased SLR coupling efficiency as well as a blueshift of the SLR resonance wavelength. At the same time, however, the geometrical increase of the circumference at the bottom of the structure reduces the strength of the LSPR leading to a less pronounced SLR peak. (3) Tilt angle: The tilt angle of the inclined structure defines the amount of symmetry breaking, which strongly affects the strength and direction of the diffractively scattered radiative field. No diffractively scattered field coupling is possible under normal-incidence illumination for upright structures (0° inclination). As the tilt angle increase, the coupling of the diffractively scattered radiative field is enhanced, resulting in a rapid formation of a strong SLR dip. However, at steeper tilt angles, the propagation direction of the scattered field becomes tilted as well, reducing the in-plane component and thus the SLR coupling efficiency, which leads to a broadening and reduced depth of the SLR dip above an optimum tilt angle, and 30° inclined structure array have complex plasmonic multi-mode generated without SLR resonance. These results not only clearly demonstrate the SLR characteristics of our system but also provide the ability to tune the resonance wavelength and strength by tailoring the geometry of the 3D inclined structures.

### 2.2 Fabrication of 3D inclined nanostructures via TEM grid–based stencil lithography

**Figure 2:**
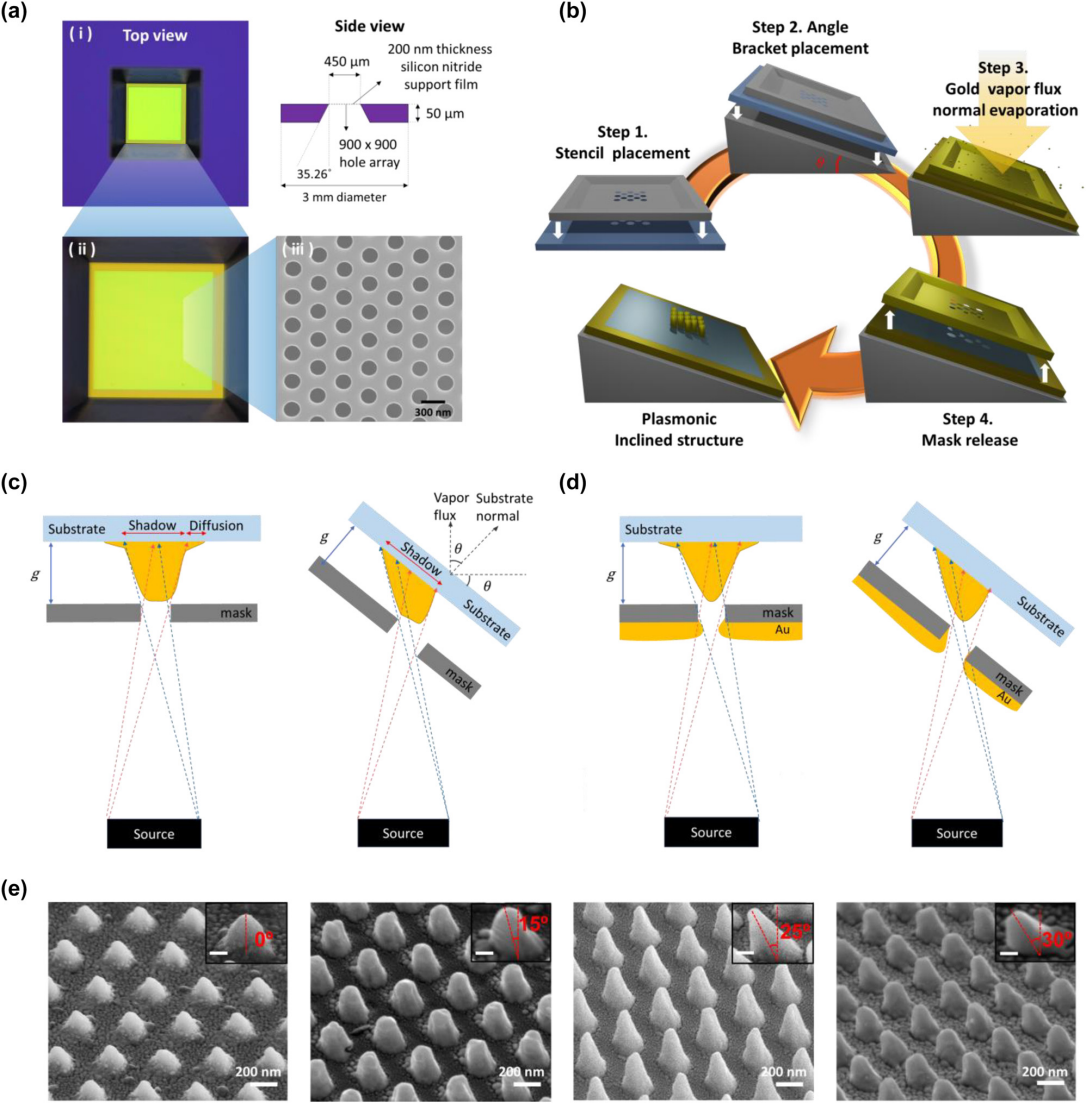
Fabrication of 3D inclined nanostructures by TEM grid–based stencil lithography. (a) Geometry of a commercial mask (TEM grid). Top view of the mask with optical microscopy (i–ii) and scanning electron microscopy (iii). (b) Fabrication process of the TEM grid–based stencil lithography with angled deposition. (c) Schematic diagram of the deposited structures affected by the shadow effect in the case of normal (left) and angled deposition (right). (d) Schematic diagram of the deposited structures affected by the blurring and clogging effect in the case of normal (left) and angled deposition (right). (e) Scanning electron microscope images of 500 nm period gold nanostructure arrays with inclination angles of 0°, 15°, 25°, and 30°. Insets show the side view of each sample with the tilted angle defined and each scale bar indicates 100 nm.

Nanostencil lithography (NSL) is a masking deposition method for patterning nanoscale structures using a mask, which simplifies the fabrication step [37]–[39]. The availability of reproducible masks is a crucial component of NSL for repetitive experiments including biosensing applications [40], [41]. In this study, we demonstrate the fabrication of 3D inclined structure arrays via stencil lithography by utilizing a commercial TEM grid as a mask. A TEM grid is a free-standing thin film of silicon nitride with (optionally) a periodic array of holes supported on a silicon wafer that is used as a carrier for thin specimens for transmission electron microscopy [42]. However, a commercial TEM grid is also ideally suited to be repurposed for NSL due to the high temperature and physical stability and low stress characteristics of the thin-film silicon nitride based hole array as well as its highly reproducible geometry [43], [44]. The TEM grid (21587-10, TED PELLA) used in this work consists of a 250 nm diameter and 500 nm period hexagonal array of holes (900 × 900) in a 200 nm thick silicon nitride film, which is supported by a 3 mm diameter silicon wafer with a 35.26° opening angle of the window (Figures 2a and S3). The fabrication process of TEM grid–based stencil lithography for inclined nanostructures is described in Figure 2b. The TEM grid is placed on a fused silica substrate while minimizing the gap between them (Step 1) and attached to an angle bracket (Step 2), which provides inclination control of the TEM grid–based stencil mask. The sample is then loaded into the deposition chamber with the substrate and stencil mask inclined with respect to the source, and gold is deposited via electron beam evaporation (DKOS-M2, DAEKI HI-TECH) with 400 nm thickness, 4 Å/s of deposition speed, and at 3 × 10^−7^ Torr of chamber pressure (Step 3). The inclined plasmonic structure array is obtained after releasing the mask (Step 4).

The deformation of the nanostructures due to blurring and clogging effects during the fabrication is an inherent limitation of NSL [45]–[49]. The blurring effect is caused by a divergence of the vapor flux and the unavoidable gap between the mask and the substrate, which produces structures larger than the aperture size and with blurred boundaries [40], [45]. The clogging effect describes the reduction of the aperture size due to the accumulation of the deposited material on the membrane and the sidewall of the aperture [48], [49]. This phenomenon reduces the effective aperture size, resulting in sloped edges of the deposited structure [50]. A schematic of the blurring and clogging effect during the normal and angled stencil lithography are shown in Figure 2c and d, respectively. Both methods result in the fabrication of 3D cone-shaped structures with blurred boundaries, a larger size than the aperture due to the blurring effect, as well as nonflat tops due to the clogging effect. For the experimental demonstration, 3D nanostructure arrays with various inclinations (0°, 15°, 25°, and 30°) were fabricated by TEM grid–based angled stencil lithography. The inclination angle of the structures was confirmed with a 60° angled scanning electron microscope measurement, which revealed a deviation of approximately 5° from the tilted angle set during the deposition (Figure 2e). The height of the fabricated nanostructures was investigated with atomic force microscopy (NX10, Park systems) measurements. The 0°, 15°, 25°, and 30° inclined nanostructures have a height of 210 nm, 290 nm, 310 nm, and 250 nm, respectively. The inevitable clogging effect in NSL results in a lower height of the structures than the deposition thickness, and the measured heights of the fabricated structures suggest that the clogging effect may decrease with increasing tilt angle of the deposition. However, at deposition angles above 30°, we observed a drastic increase of blurring and clogging effects as well as a sudden decrease in the reproducibility of the fabricated structures, likely caused by the geometric obstruction of the mask holes at high tilt angles due to the 200-nm silicon nitride film thickness. As a result, the 30° sample shows random depositions of vapor flux on the substrate surface and a less sharp shape as well as a lower height of the structure.

### 2.3 Optical characterization of the fabricated nanostructure arrays

**Figure 3:**
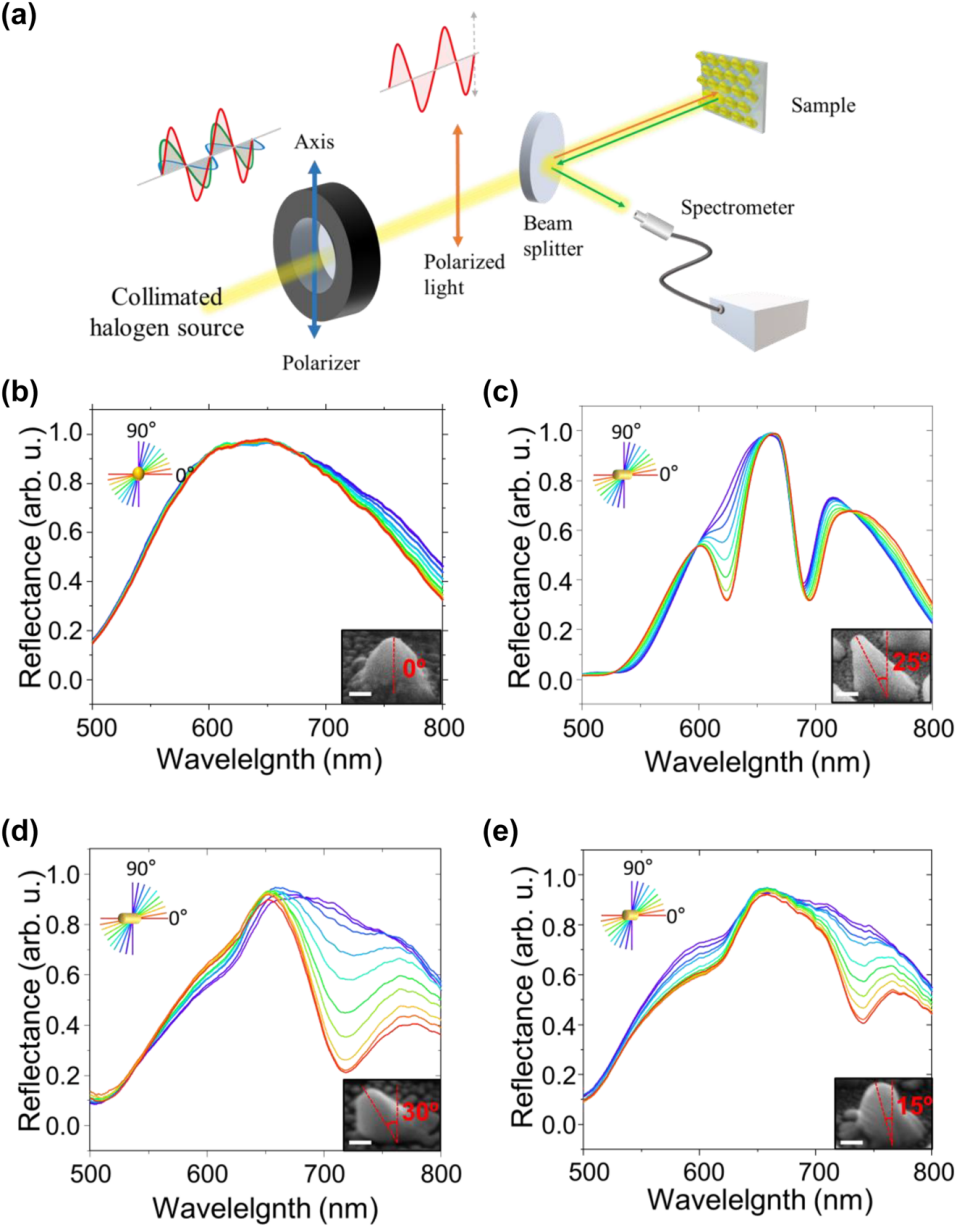
Optical characterization of the fabricated inclined nanostructure arrays. (a) Schematic diagram of the experimental setup. Polarization-resolved reflectance spectra of the 500-nm period gold nanostructure arrays with inclination angles of (b) 0°, (c) 25°, (d) 30° and (e) 15°. Insets show the side view of each sample with the inclination angle as a guide to the eye; the scale bar indicates 100 nm. A polarization axis parallel to the inclination direction is defined as 0°.

The optical properties of the fabricated inclined nanostructure arrays were characterized by using the experimental setup presented in Figure 3a. The polarization of a collimated halogen light source (FOK-150W, Fiber Optic Korea) was controlled with a linear polarizer. The polarized light was used to illuminate the fabricated nanostructures, and the reflected light was measured in normal incidence via a beam splitter with a spectrometer (HR4000, Ocean Optics). The reflection spectra from the nanostructures were recorded for different linear polarization states of the incident light. The reflection spectrum from 0° (noninclined) nanostructures shows no resonance dip and polarization switching characteristics, indicating that no plasmonic resonance occurs at these nanostructures over the measured 500 nm–800 nm wavelength range (Figure 3b). On the other hand, the reflection spectrum from 25° nanostructures shows clear resonance dips of polarization-sensitive SLR and polarization in-sensitive LSPR (Figure 3c). Additionally, the reflection spectra of the 15° and 30° nanostructures are shown in Figure 3d and e, respectively, which exhibit significantly broadened SLR resonance characteristics and lack any LSPR resonance within the measured wavelength range. The observed reflection spectrum is related to the surface morphology of the respective nanostructure sample. The SLR can be induced in periodic inclined structure arrays with a broad range of nonideal shapes and the resonance width is dictated by the structure’s height and how well defined the shape is. In contrast, the LSPR is only present when the blurring effect is minimized and the boundary between the structure and the substrate is clearly distinguished.

To explore the complex underlying geometry-related plasmonic interactions within the inclined structure, we numerically calculated the field distribution and reflection spectra of inclined structures with rounded tips and trapezoid shapes (Figures S4–11), which are more representative of the structures fabricated with our NSL approach. For a rounded-tip structure, the LSPR wavelength is unchanged and the SLR is slightly blue-shifted and reduced because of decreased coupling efficiency and mode volume. A trapezoid shape of the structure shows a more dramatic decrease of the SLR coupling efficiency than a rounded-tip structure, because an increase of the trapezoid ratio affects the propagation direction of the diffractively scattered radiative field relative to the plane of the array. As the trapezoid ratio increases, we observe a blueshift of the SLR and a redshift of the LSPR wavelength with a crossing point around 10 % of trapezoid ratio where the LSPR becomes suppressed. These results demonstrate a very sensitive dependence of the plasmonic resonance wavelengths and shapes on the exact geometry of the structure, which is difficult to control in the fabrication process. Hence, we suggest that the absence of LSPR modes for the 15° and 30° structures is due to geometry-related spectral shift (also affecting the SLR position) and the blurring effect of the circular bottom boundary of the structure during the fabrication process. The quality factor (*Q*-factor) is a useful parameter to evaluate the efficiency of the plasmonic resonance system, defined as *Q* = *λ*_resonance_/Δ*λ*_FWHM_, where *λ*_resonance_ and Δ*λ*_FWHM_ are the center wavelength and full-width at half maximum (FWHM) of the plasmonic resonance, respectively [51], [52]. For most LSPRs, the *Q*-factors are found to be *Q* < 10 due to the intrinsic ohmic losses present in metals at optical frequencies [53]. On the other hand, the collective resonance of many nanostructures arranged in an array leads to a much narrower linewidth of the SLR than that of the LSPR, resulting in a higher *Q*-factor. For the 25° inclined nanostructures, the resonance wavelength is 623 nm while the FWHM is 30 nm in the ambient air environment, yielding a *Q*-factor of ∼20.

**Figure 4:**
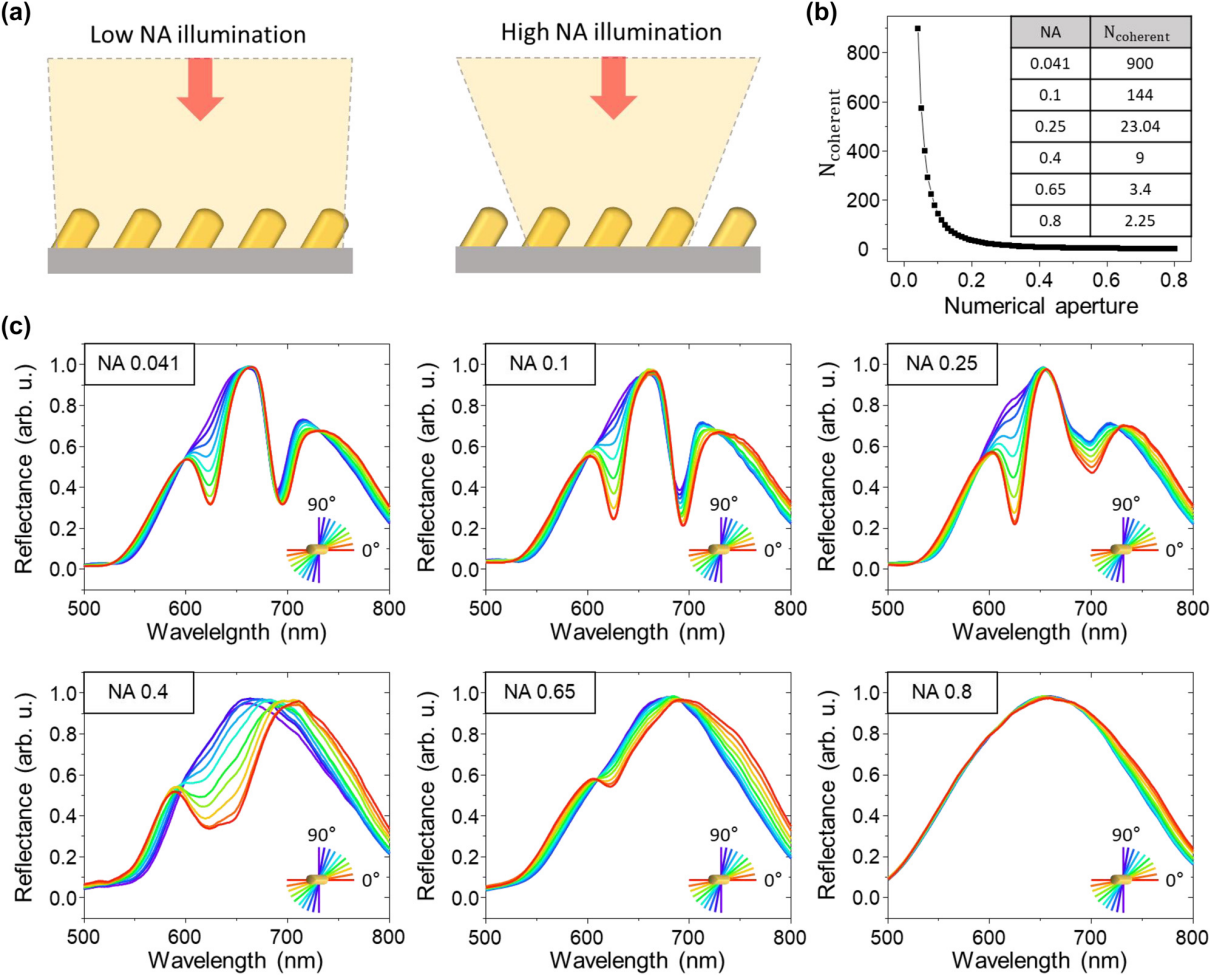
Effect of spatial coherence of the incident light on the SLR efficiency. (a) Illustration of the incident light onto the inclined nanostructure array under low NA and high NA conditions. (b) Number of coherently illuminated structures as a function of NA for 600 nm of resonance wavelength and 500 nm periodicity of the 2D array. (c) Reflectance spectra as a function of linear polarization change for a NA of 0.041, 0.1, 0.25, 0.4, 0.65, and 0.8.

The SLR effect can be efficiently induced when the scattered radiative fields from a sufficient number of individual nanostructures are in-phase within the array [54]. The spatial coherence of the incident light determines the phase of the scattered radiative field at each nanostructure contributing to coherent diffraction [55]. With increasing numerical aperture of the incident light, the spatial coherence decreases, which limits the number of modes that can interact in-phase with neighboring structures (Figure 4a) [56], [57]. Previous SLR studies have observed that a lower NA induces SLR more efficiently, and that SLR cannot be generated under illumination conditions with NA > 0.5. The wavelength *λ* of the focused incident light has a transverse spatial coherence on the order of *λ*/NA, and the number of coherently illuminated structures for a collective resonance within a 2D periodically arranged array is given by

$${\text{N}}_{\text{coherent}}={\left(\lambda_{\text{resonance}}/\left(\text{NA}\cdot \alpha \right)\right)}^{2},$$

where *λ*_resonance_ is the wavelength of the SLR and *α* is the periodicity of the 2D array [9], [58]. The number of coherent structures as a function of NA was calculated with a resonance wavelength of 600 nm and a periodicity of the 2D array of 500 nm (Figure 4b). To experimentally demonstrate how changing the numerical aperture of the illumination light affects the SLR (Figure 4c), we observed the reflection spectra with polarization switching characteristics of the SLR to confirm the lattice resonance effect. For NA = 0.041 to NA = 0.25, the FWHM of the resonance dip is unaffected, but increases significantly above NA = 0.4, and no more lattice effect could be observed above NA = 0.65. Additionally, we investigate the far-field diffraction properties of our asymmetric structures and their potential effect on the measurements. The diffraction angle *θ*_*d*_ can be described as

$$n_{1}\left(\sin\theta_{i}\pm \sin\theta_{d}\right)=m\lambda /\alpha ,$$

where *n*_1_, *θ*_*i*_, *m*, and *α* are the refractive index of the surrounding medium, the incidence angle of the illuminating light, the diffraction order, and the periodicity, respectively [33]. Using our experimental parameters (*n*_1_ = 1, *θ*_*i*_ = 0, *d* = 500 nm) results in |*θ*_*d*_| > 90° for illumination wavelengths above 500 nm. Hence, the measurements of the reflection spectra under normal-incidence illumination are not affected by optical grating effects in the observed wavelength range.

### 2.4 Environment sensitivity of 3D inclined structure arrays

Label-free optical biosensing monitors the refractive index changes accompanying biological binding events when target biological molecules react with receptors that are immobilized on the surface [59], [60]. The local refractive index at the surface rises during a biological binding event because the refractive index of most biological molecules (*n*_bio_ = 1.45–1.55) is larger than that of water (*n*_water_ = ∼1.33) [4]. The shift of the plasmon resonance with environmental refractive index change can be analyzed via electrostatic model. The increase of refractive index around the plasmonic structure during the biological binding event induce the red shift of plasmonic resonance, because the increase of dielectric function induces the increase of electric dipole moment, which reduces the restoring electric force of charge [61]. The environmentally highly sensitive characteristics of plasmonic techniques are, therefore, suitable for detecting such biological binding events [62]. The refractive index sensitivity and figure of merit (FOM) parameters have been used to evaluate the performance of LSPR and SLR systems for biological applications [63]. The refractive index sensitivity and FOM are defined as (Δ*λ*_shift_/Δ*n*) and (Δ*λ*_shift_/Δ*n*)(1/Δ*λ*_FWHM_), respectively, where Δ*λ*_shift_ is the shift of the resonance wavelength for a Δ*n* change of the refractive index [64].

**Figure 5:**
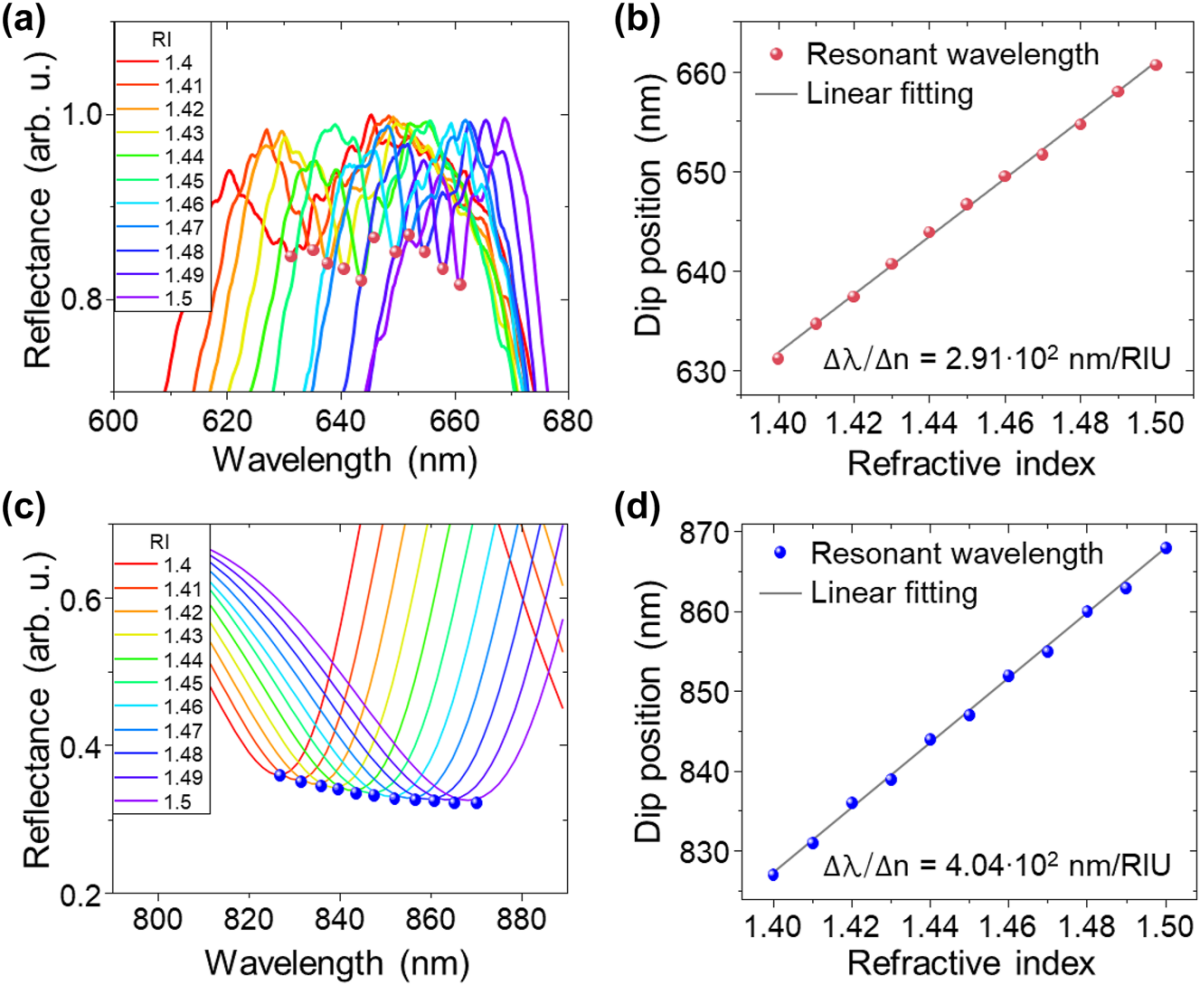
Environment sensitivity of the lattice plasmon resonance from 3D inclined nanostructures. (a) Measured reflectance spectra from 3D inclined nanostructures for varying refractive index values of the analyte with parallel polarization of the illuminating light with respect to the inclination direction of the structures. RI: refractive index. (b) Linear relationship between the refractive index and the measured dip position of the resonance wavelength. (c) Numerically calculated reflectance spectra of 3D inclined nanostructures for varying refractive index values with parallel polarization of the illuminating light with respect to the inclination direction of the structures. (d) Linear relationship between the refractive index and the calculated dip position of the resonance wavelength.

The optical biosensing performance of the 3D inclined nanostructure array fabricated by our NSL technique is determined by demonstrating its sensitivity to refractive index changes. We recorded reflection spectra from the 25° inclined structure array (Figure 3c) under NA = 0.041 normal-incidence illumination while applying a refractive index oil (Series A and AA, Cargile) onto the structure array within a range of *n* = 1.4 to *n* = 1.5 in steps of Δ*n* = 0.01 ± 0.0002 (Figure 5a). The resonance dip wavelength of the reflectance spectra is clearly red-shifted with increasing refractive index. The full-range reflection spectra are shown in Figure S8. The relationship between the refractive index and the resonance wavelength is shown in Figure 5b, yielding a refractive index sensitivity of our structure array of 291 nm/RIU with a highly linear resonance wavelength shift (R-square value of 0.99872). Additionally, a FOM of ∼44.7 and a *Q*-factor of ∼101.6 were obtained at the *n* = 1.5 refractive index environment (*λ*_resonance_ = 661 nm, Δ*λ*_FWHM_ = 6.5 nm). For validation, we also numerically calculated the refractive index sensitivity of the inclined structure array as a function of the refractive index from *n* = 1.4 to *n* = 1.5 with Δ*n* = 0.01 steps (Figure 5c), yielding a refractive index sensitivity of 404 nm/RIU (Figure 5d) with a highly linear redshift of the resonance wavelength (R-square value of 0.99846). This result demonstrates approximately 10 times higher sensitivity than most LSPR system and is comparable with other SLR system (Supplementary Table 1).

## 3 Conclusions

In this study, we demonstrated plasmonic lattice coupling at 3D inclined nanostructure arrays fabricated with commercial TEM grid–based stencil lithography and their potential for biosensing applications with a high *Q*-factor, FOM, and refractive index sensitivity. Our results show a high performance of the metasurfaces for environmental sensitivity with a simple fabrication process. We have found that the 3D symmetry breaking of an inclined nanostructure array induces a large mode volume facilitating strong diffractively scattered radiative coupling between neighboring structures, which is in excellent agreement with numerical calculations. Additionally, the role of spatial coherence in the SLR effect, which has been suggested in previously reported SLR studies, has been experimentally confirmed. The proposed technology is expected to be of great use in the development of SLR-based metasurfaces and nanophotonic applications including label-free optical biosensing [65], [66].

## Supplementary Material

Supplementary Material Details
