# Supplementary material for: Three-dimensional surface lattice plasmon resonance effect from plasmonic inclined nanostructures via one-step stencil lithography
Source: Nanophotonics. 2024 Jan 12;13(7):1169–80. doi: 10.1515/nanoph-2023-0755 (PMC11501154; doi:10.1515/nanoph-2023-0755)
Supplement: Supplementary file 1 — Supplementary Material Details [file j_nanoph-2023-0755_suppl_001.pdf]

Supplementary Material

Three-dimensional surface lattice plasmon resonance effect from plasmonic inclined nanostructures via one-step stencil lithography

Tae-In Jeong[1,†], Sehyeon Kim[1,†], San Kim[1], Minchan Shin[1], Alexander Gliserin[1,2], Tae Young Kang[1], Kyujung Kim[1,2], Seungchul Kim[1,2]*

†Tae-In Jeong and Sehyeon Kim contributed equally to this work.

[1] Department of Cogno-Mechatronics Engineering, College of Nanoscience and Nanotechnology, Pusan National University, Busan 46241, Republic of Korea

[2] Department of Optics and Mechatronics Engineering, College of Nanoscience and Nanotechnology, Pusan National University, Busan, 46241, Republic of Korea

Corresponding Author: Seungchul Kim (s.kim@pusan.ac.kr)

Keywords: surface lattice plasmon resonance; stencil lithography; TEM grid; nanophotonics

Mechanism	*Q*	*λ* (nm)	Materials	Fabrication method	Light source	Reference
SLR	101	661	Inclined nanostructures	NSL	Halogen lamp	This work*
LSPR	<10	700	Au NPs	EBL	Tungs.-halogen lamp	1
LSPR	<10	610	Au nanorods	EBL	Xenon lamp	2
LSPR	<10	598	Au NPs	EBL	N/A	3
LSPR	8	N/A	Al NPs	EBL	EELS	4
LSPR	9	N/A	Au NPs	EBL	Ellipsometer	5
SLR	19	843	Au NPs	EBL	Ellipsometer	5
SLR	25	930	Au NPs	EBL	Collimated source	6
SLR	30	850	Au NPs	EBL	Tungs.-halogen lamp	1
SLR	40-60	600	Au NPs	EBL	Ellipsometer	7
SLR	60	800	Au NPs	EBL	Tungs.-halogen lamp	8
SLR	150	764	Au NPs	EBL	Tungs.-halogen lamp	9

Q quality factor, λ resonance wavelength , *SLR* surface lattice resonance, *LSPR* localized surface plasmon resonance,
NSL nano stencil lithography, *EBL* electron beam lithography, *EELS* electron energy loss spectroscopy.
* *Q* of this work was obtained in an environment with refractive index of n=1.50.

Supplementary Table 1. Summary of experimentally obtained Q-factors of SLR and LSPR with different fabrication methods.

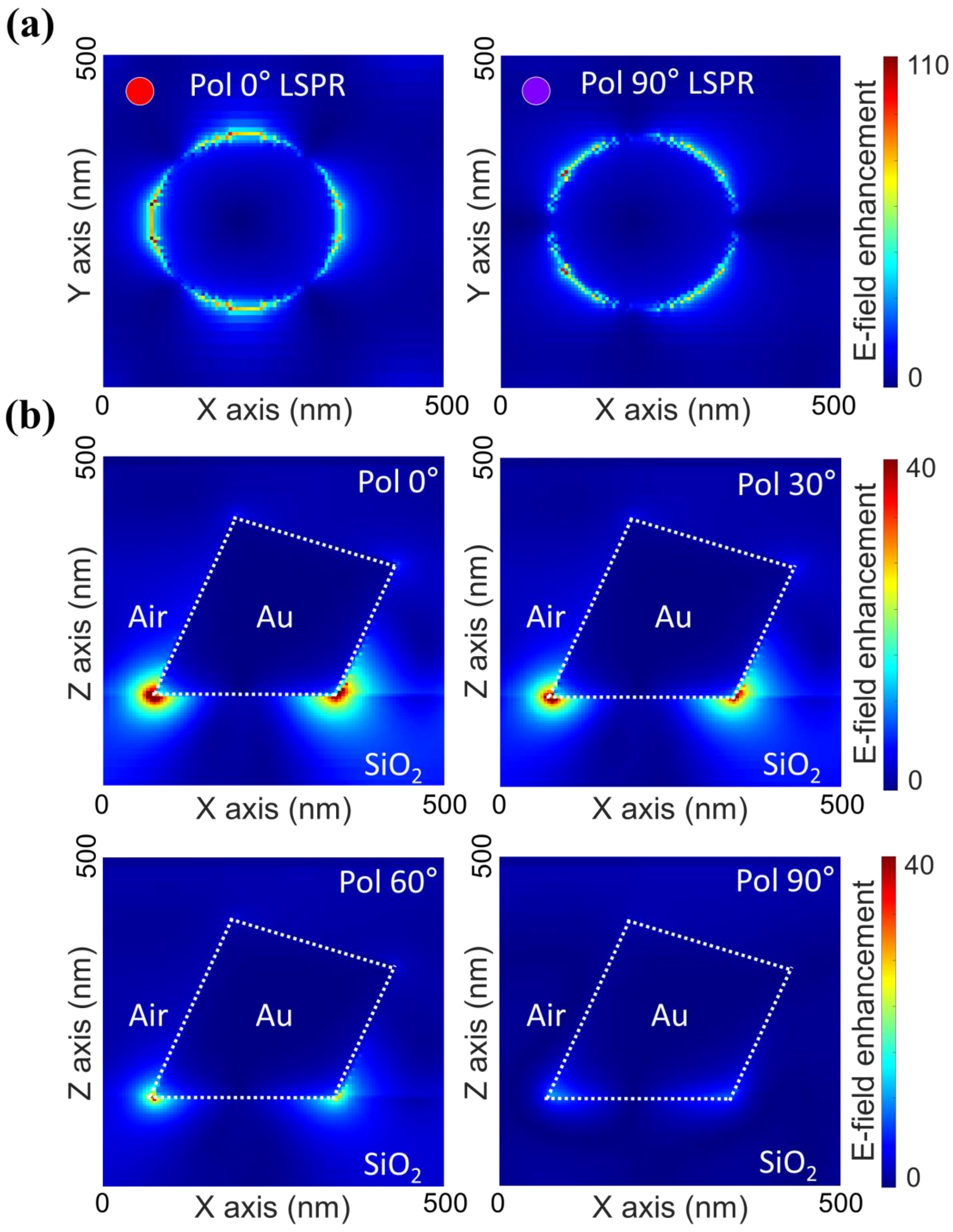

Supplementary Figure 1. (a) Numerically calculated electric field distribution at the boundary between the structure and the substrate and (b) a cross-section through the center of the pillar with different linear polarizations at 653 nm wavelength.

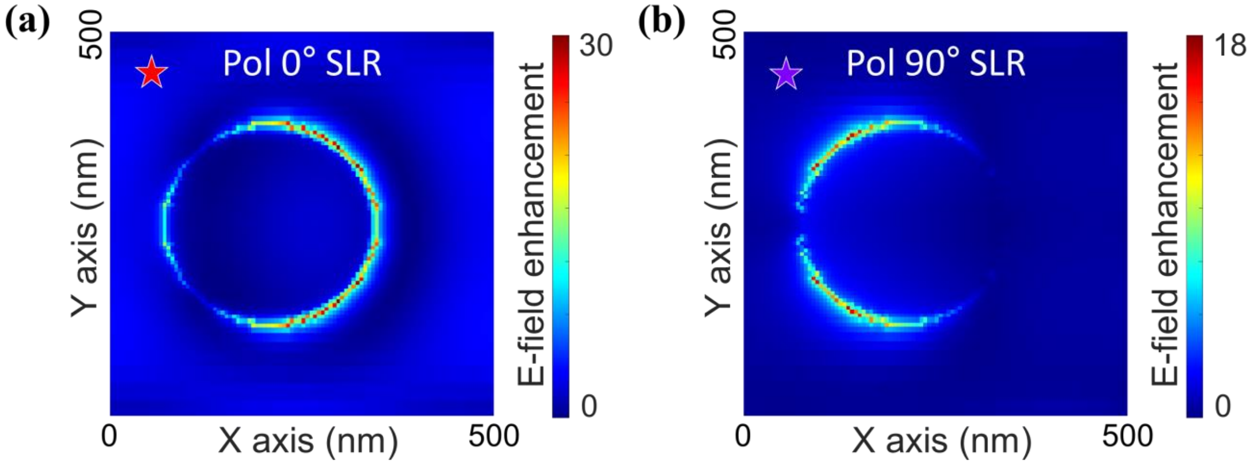

Supplementary Figure 2. Numerically calculated electric field distribution at the boundary between the structure and the substrate with different linear polarizations at 690 nm wavelength.

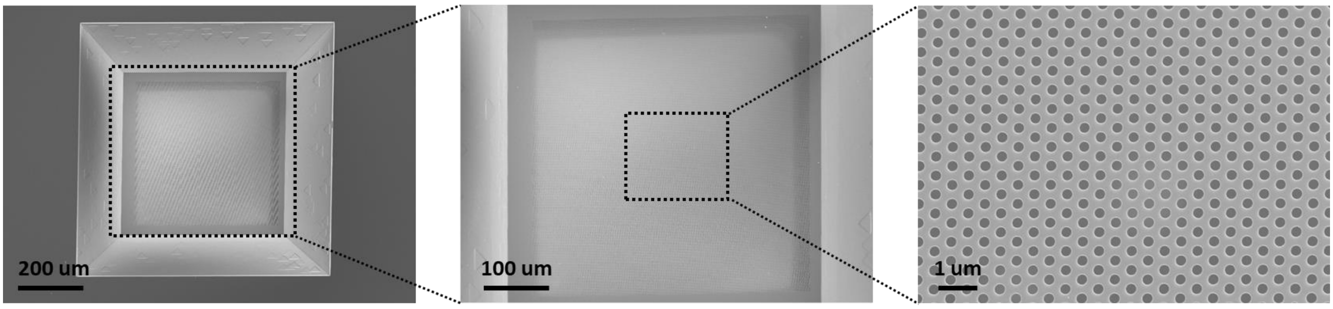

Supplementary Figure 3. Scanning electron microscopy image of a TEM grid, which is utilized as a mask for stencil lithography.

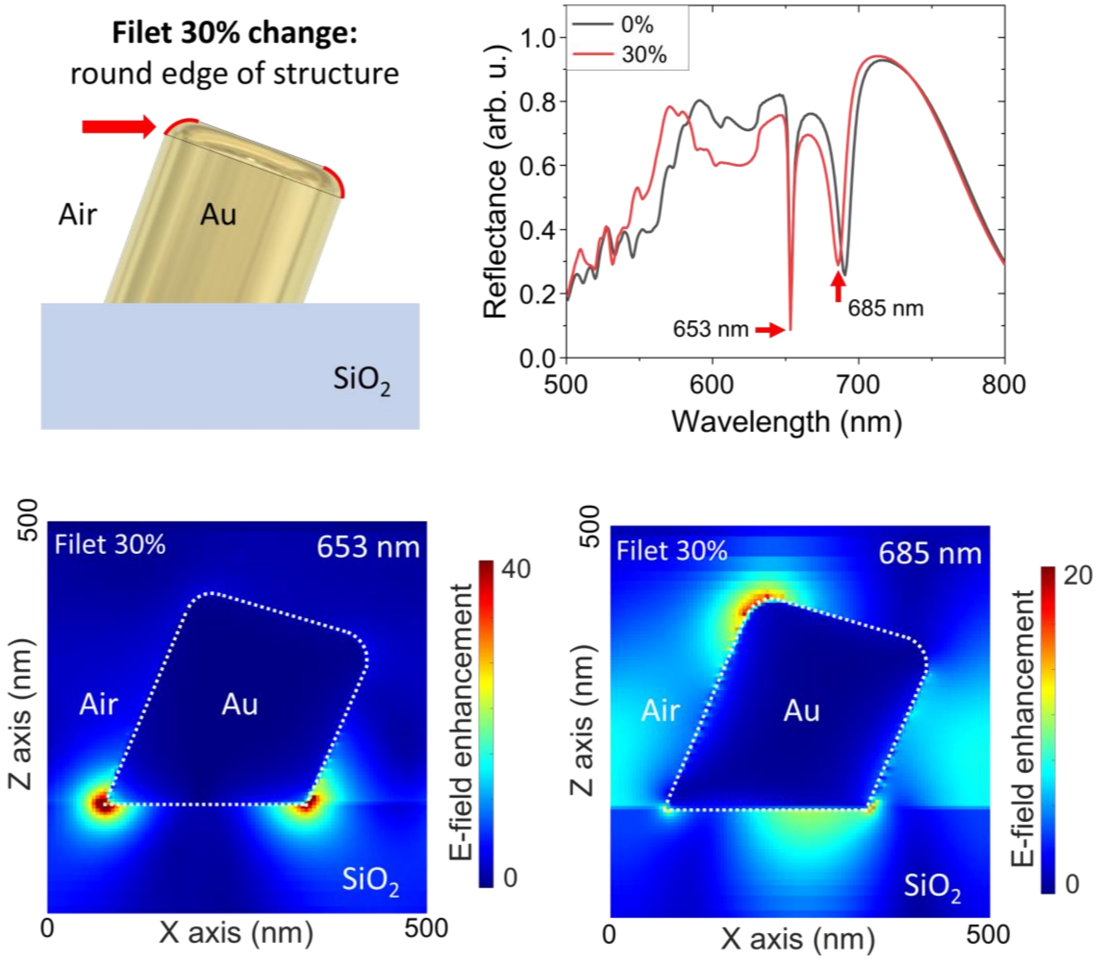

Supplementary Figure 4. Numerically calculated reflectance spectra and electric field distribution at different wavelengths from an inclined structure with a rounded edge (the radius of the filet is 37.5 nm) under normal-incidence illumination with linear polarization. Note that the white dotted line shows the outline of rounded-edge inclined structure.

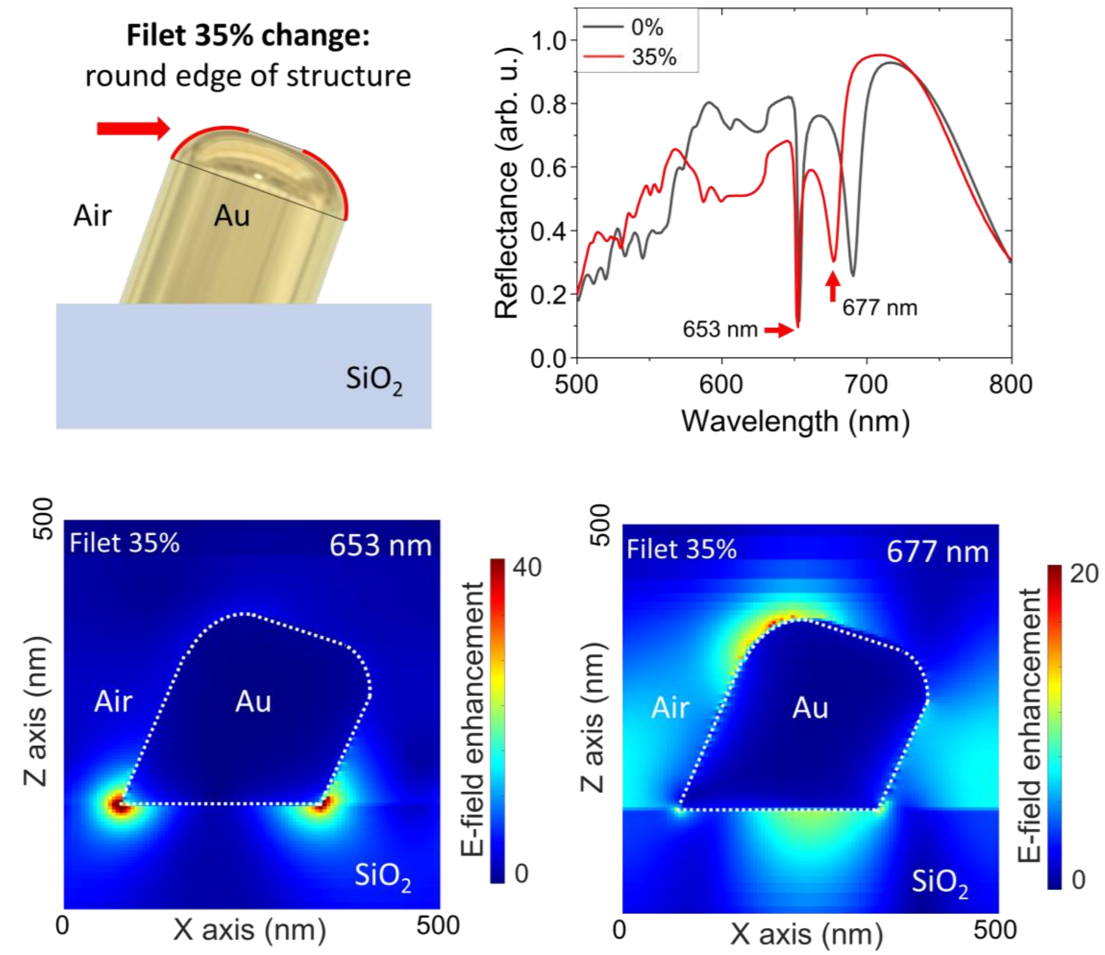

Supplementary Figure 5. Numerically calculated reflectance spectra and electric field distribution at different wavelengths from an inclined structure with a rounded edge (the radius of the filet is 43.75 nm) under normal-incidence illumination with linear polarization. Note that the white dotted line shows the outline of rounded-edge inclined structure.

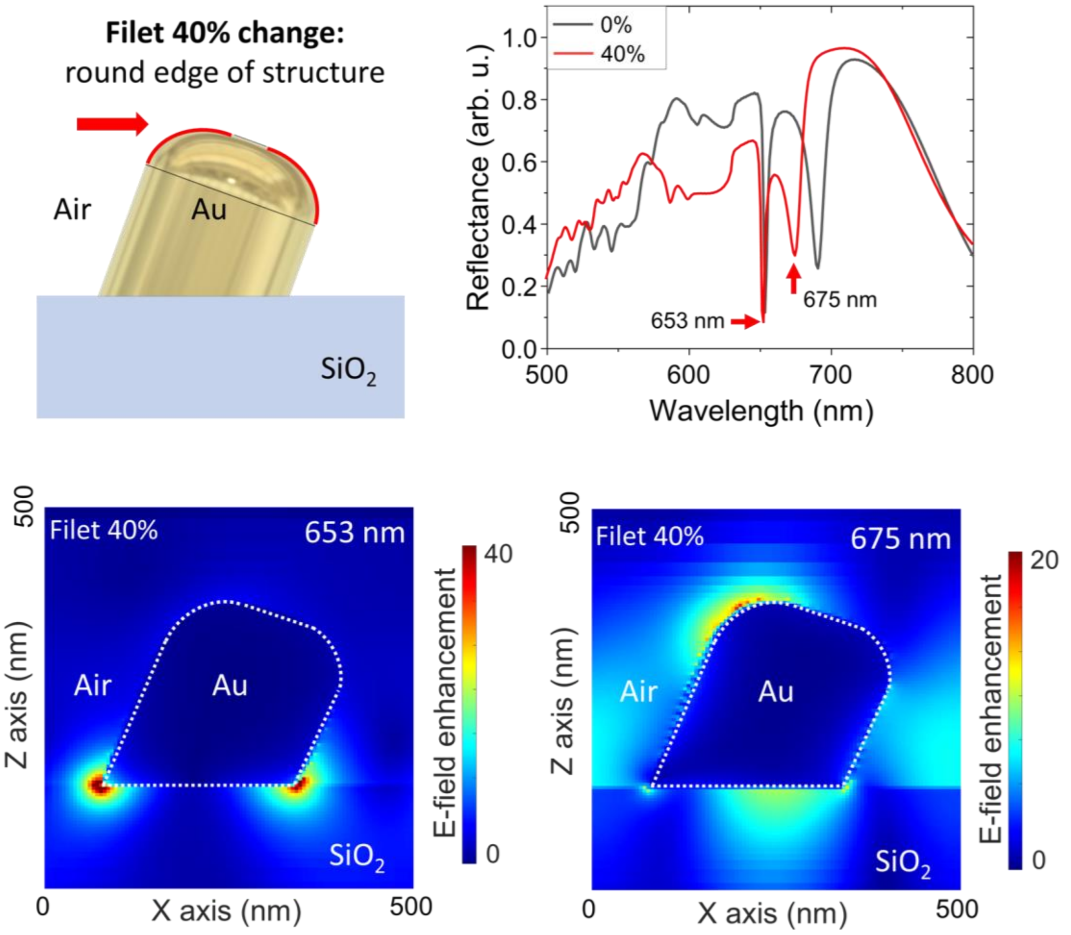

Supplementary Figure 6. Numerically calculated reflectance spectra and electric field distribution at different wavelengths from an inclined structure with a rounded edge (the radius of the filet is 50 nm) under normal-incidence illumination with linear polarization. Note that the white dotted line shows the outline of rounded-edge inclined structure.

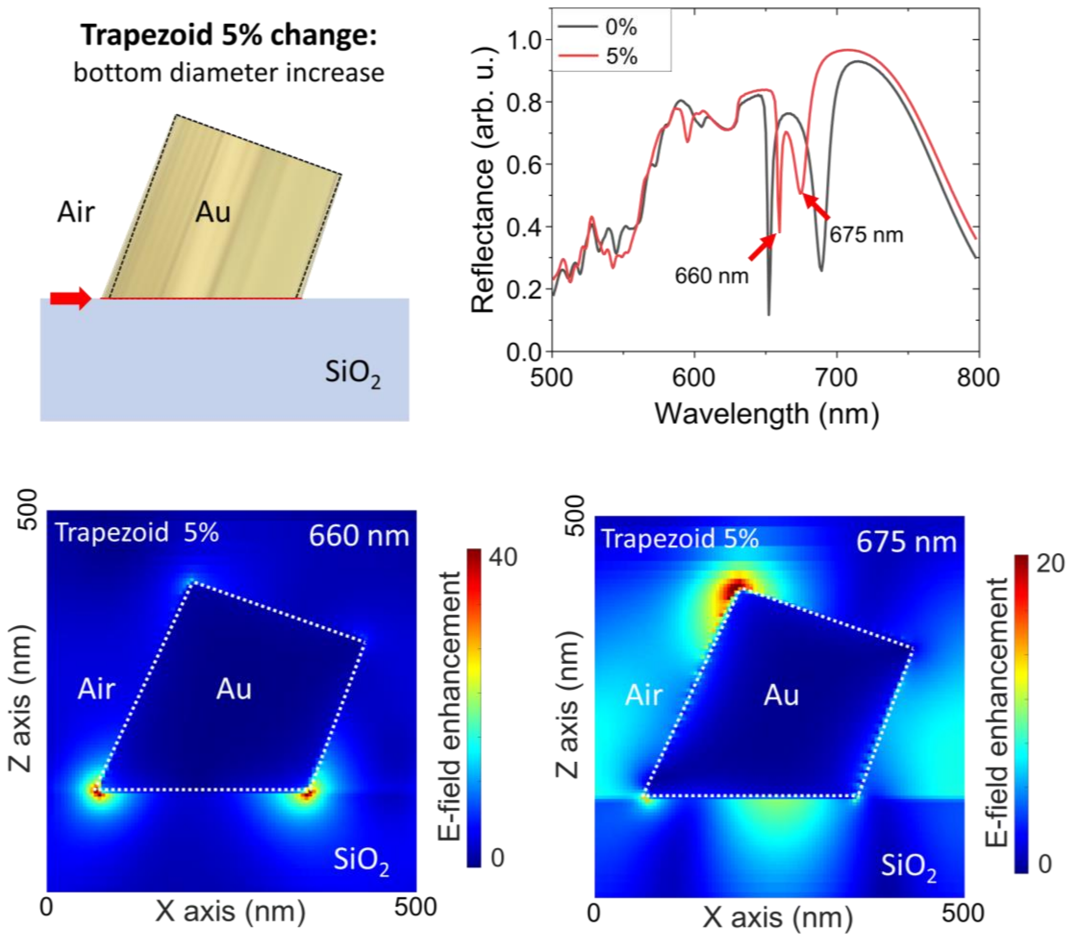

Supplementary Figure 7. Numerically calculated reflectance spectra and electric field distribution at different wavelengths from a 5% trapezoid shape (top diameter 250 nm and bottom diameter 262.5 nm) inclined structure under normal-incidence illumination with linear polarization. Note that the white dotted line shows the outline of the trapezoid inclined structure.

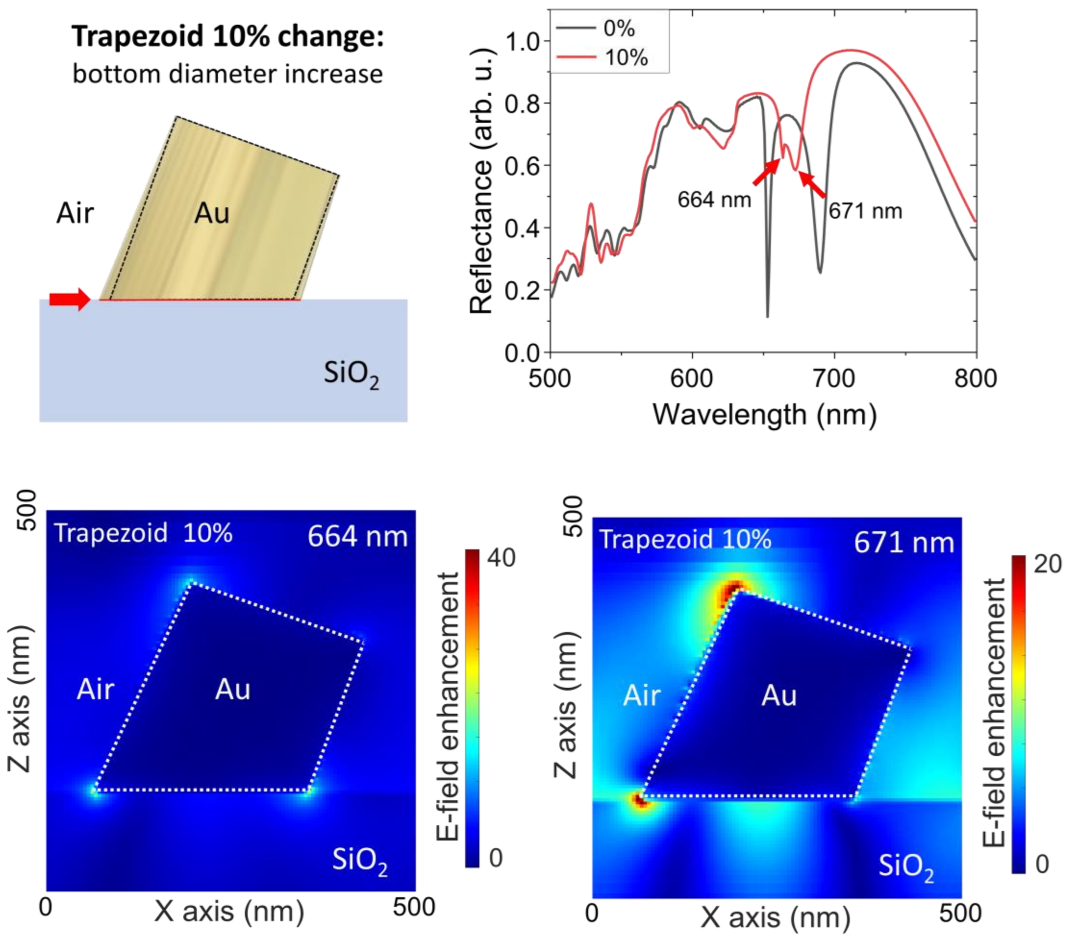

Supplementary Figure 8. Numerically calculated reflectance spectra and electric field distribution at different wavelengths from a 10 % trapezoid shape (top diameter 250 nm and bottom diameter 275 nm) inclined structure under normal-incidence illumination with linear polarization. Note that the white dotted line shows the outline of the trapezoid inclined structure.

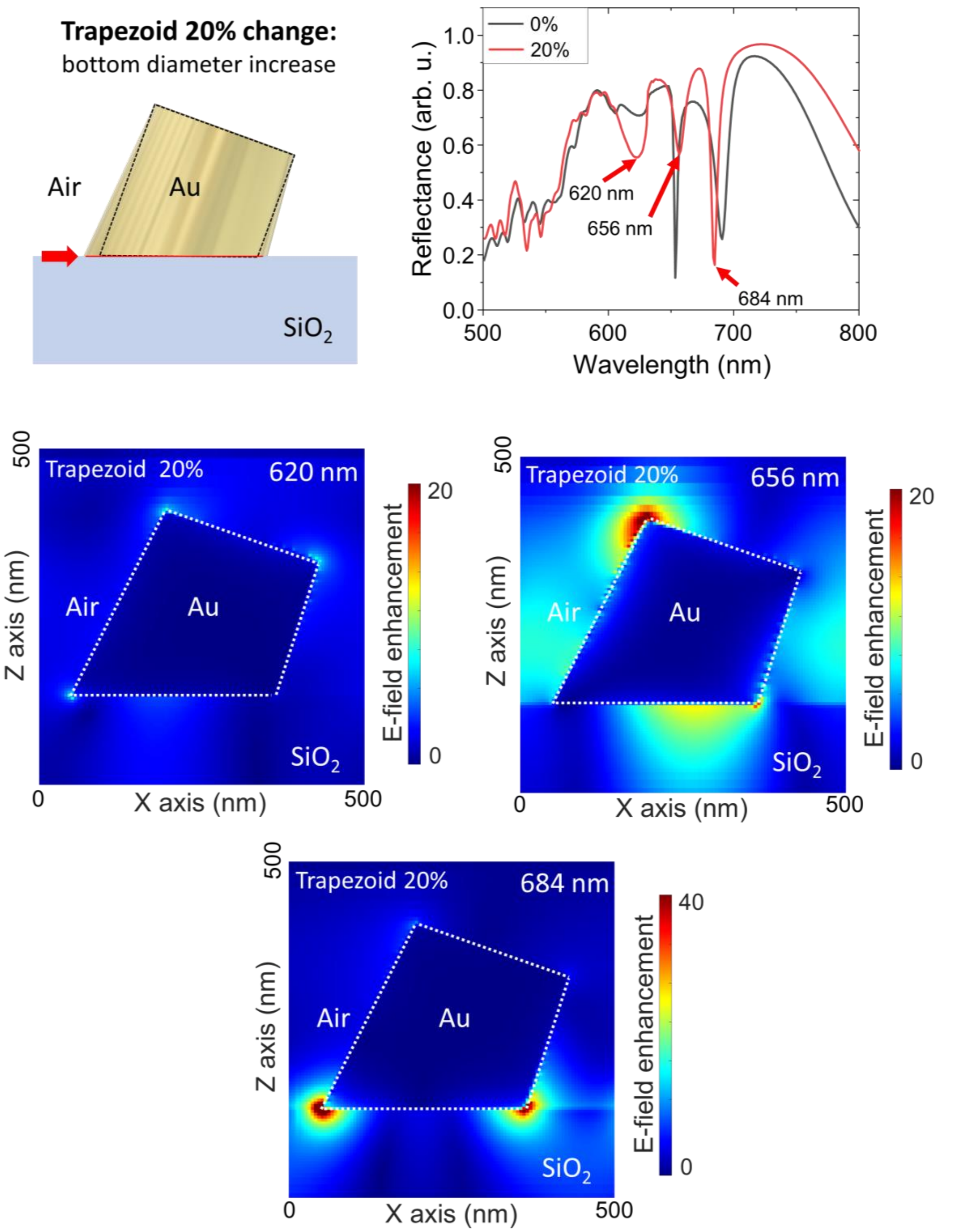

Supplementary Figure 9. Numerically calculated reflectance spectra and electric field distribution at different wavelengths from a 20% of trapezoid shape (top diameter 250 nm and bottom diameter 300 nm) inclined structure under normal-incidence illumination with linear polarization. Note that the white dotted line shows the outline of the trapezoid inclined structure.

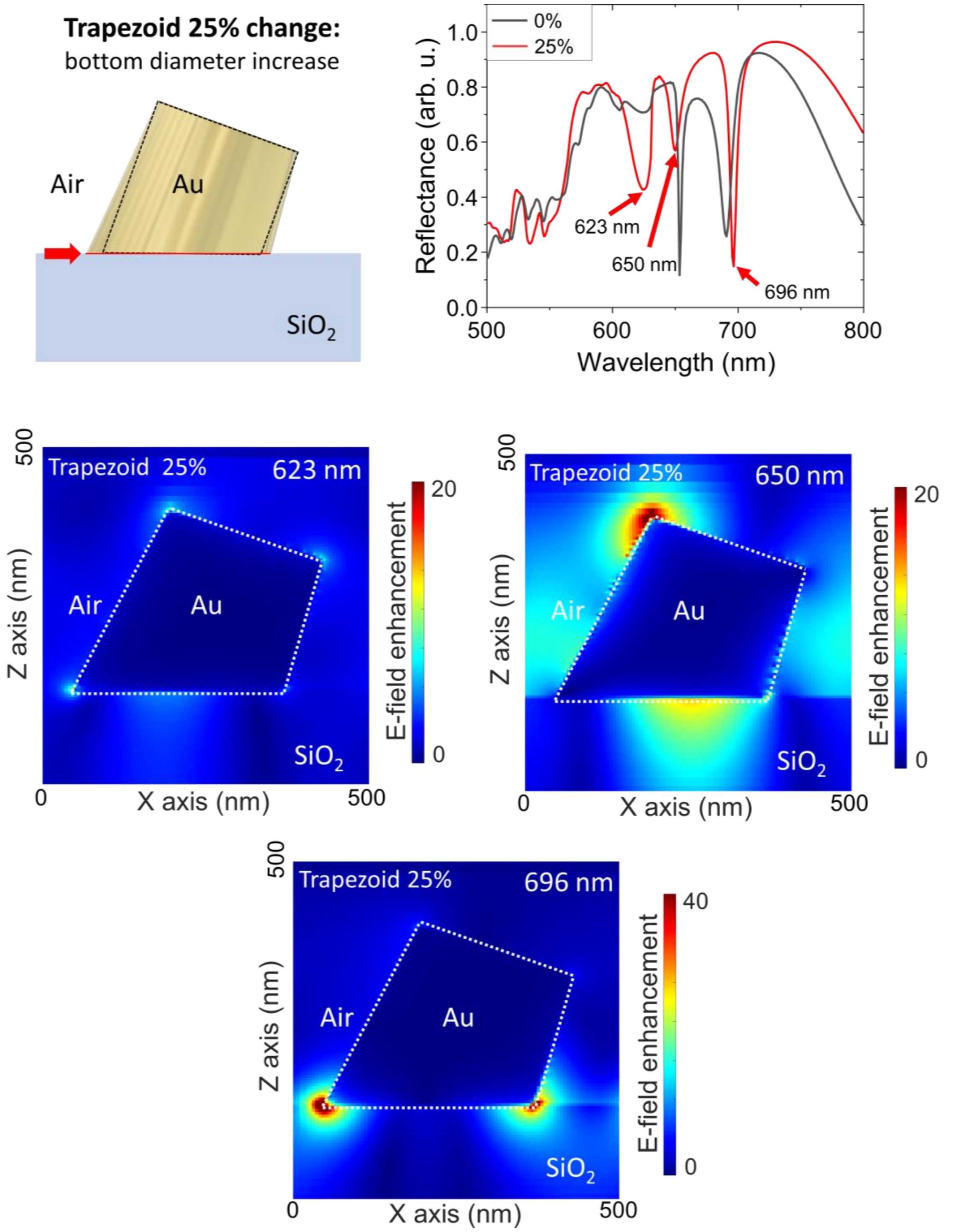

Supplementary Figure 10. Numerically calculated reflectance spectra and electric field distribution at different wavelengths from a 25% of trapezoid shape (top diameter 250 nm and bottom diameter 312.5 nm) inclined structure under normal-incidence illumination with linear polarization. Note that the white dotted line shows the outline of the trapezoid inclined structure.

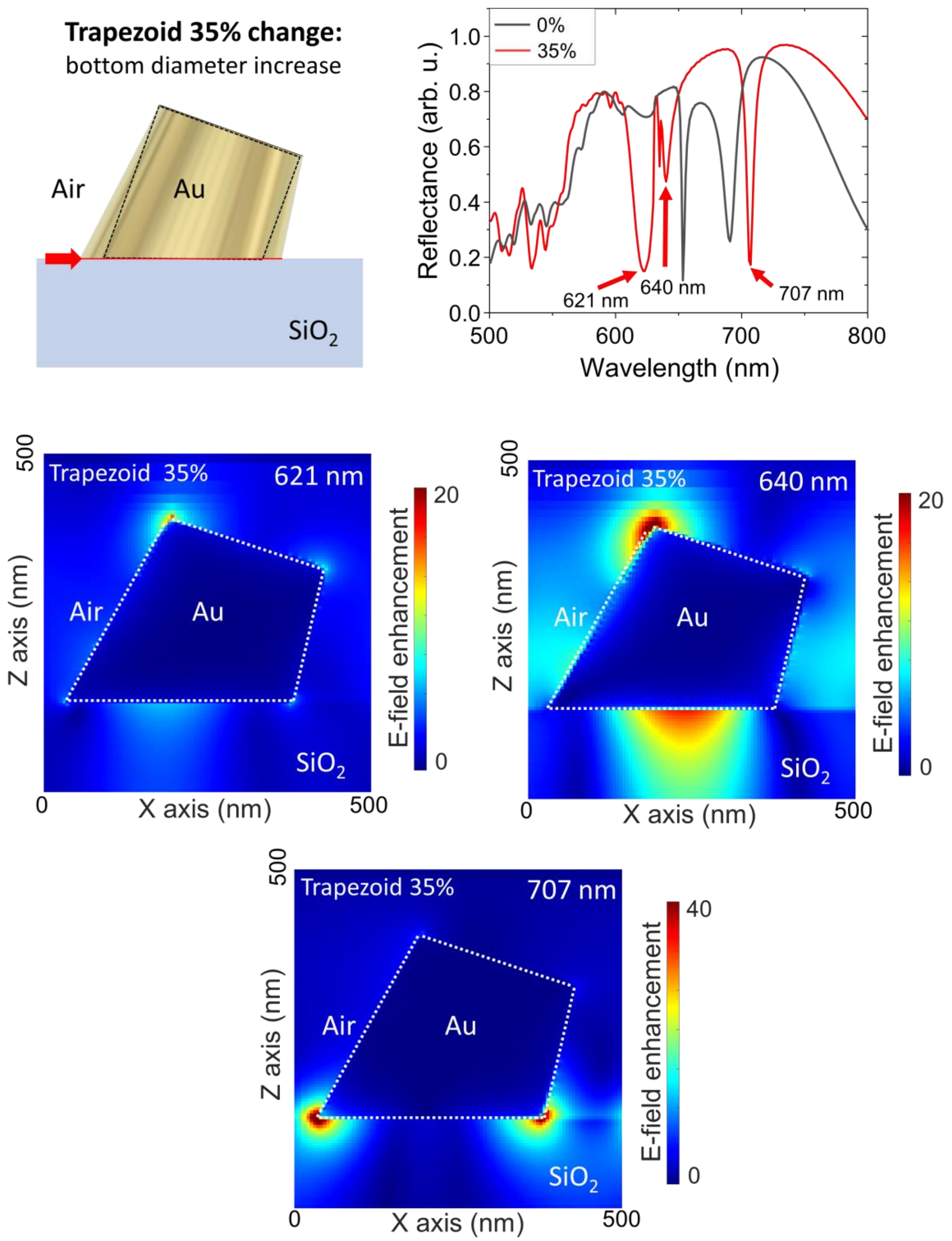

Supplementary Figure 11. Numerically calculated reflectance spectra and electric field distribution at different wavelengths from a 35% of trapezoid shape (top diameter 250 nm and bottom diameter 337.5 nm) inclined structure under normal-incidence illumination with linear polarization. Note that the white dotted line shows the outline of the trapezoid inclined structure.

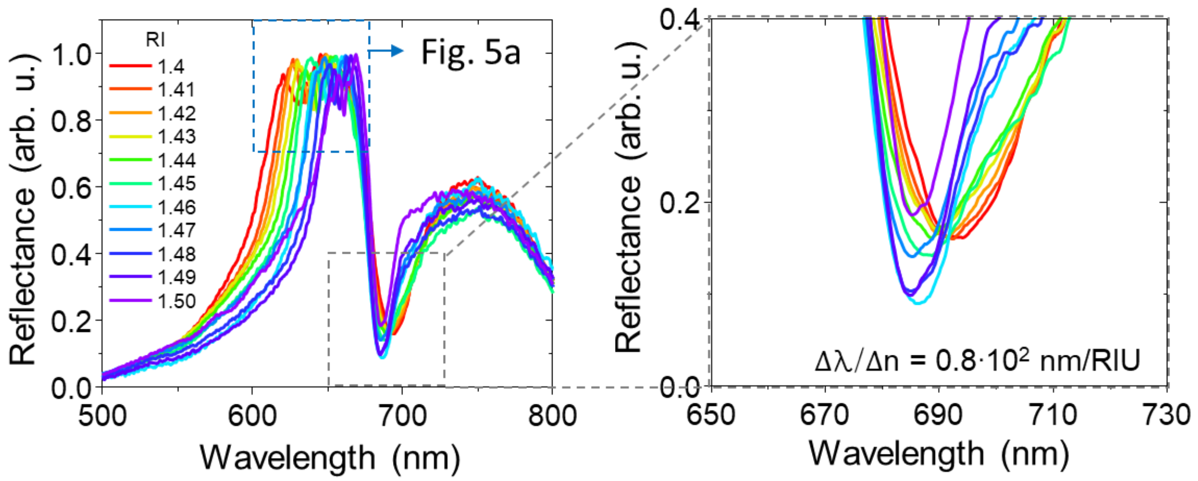

Supplementary Figure 12. Environment sensitivity of the lattice plasmon resonance from the 3D inclined nanostructure array, showing the SLR wavelength shift range (blue dashed box) and LSPR wavelength shift range (gray dashed box and inset). RI: refractive index. A multi-mode LSPR is observed with 80 nm/RIU of refractive index sensitivity.

Table references

1) Rodriguez, Said Rahimzadeh Kalaleh, et al. "Coupling bright and dark plasmonic lattice resonances." Physical Review X 1.2 (2011): 021019.
2) Si, Guangyuan, et al. "Reflective plasmonic color filters based on lithographically patterned silver nanorod arrays." Nanoscale 5.14 (2013): 6243-6248.
3) Grigorenko, A. N., et al. "Nanofabricated media with negative permeability at visible frequencies." Nature 438.7066 (2005): 335-338.
4) Martin, Jérôme, et al. "High-resolution imaging and spectroscopy of multipolar plasmonic resonances in aluminum nanoantennas." Nano Letters 14.10 (2014): 5517-5523.
5) Thackray, Benjamin D., et al. "Narrow collective plasmon resonances in nanostructure arrays observed at normal light incidence for simplified sensing in asymmetric air and water environments." ACS Photonics 1.11 (2014): 1116-1126.
6) Chu, Yizhuo, et al. "Experimental observation of narrow surface plasmon resonances in gold nanoparticle arrays." Applied Physics Letters 93.18 (2008).
7) Kravets, V. G., F. Schedin, and A. N. Grigorenko. "Extremely narrow plasmon resonances based on diffraction coupling of localized plasmons in arrays of metallic nanoparticles." Physical Review Letters 101.8 (2008): 087403.
8) Auguié, Baptiste, and William L. Barnes. "Collective resonances in gold nanoparticle arrays." Physical Review Letters 101.14 (2008): 143902.
9) Kravets, V. G., et al. "Sensitivity of collective plasmon modes of gold nanoresonators to local environment." Optics Letters 35.7 (2010): 956-958.
